# Does 3-dimensional facial attractiveness relate to golden ratio, neoclassical canons, ‘ideal’ ratios and ‘ideal’ angles?

**DOI:** 10.1186/s40902-022-00358-2

**Published:** 2022-09-07

**Authors:** Roger A. Zwahlen, Alexander T. H. Tang, Wai Keung Leung, Su Keng Tan

**Affiliations:** 1 Discipline of Oral and Maxillofacial Surgery, Faculty of Dentistry, University of Hong Kong, Hong Kong SAR, People’s Republic of China; 2 Unità Di Chirurgia Maxillofacciale E Orale, Ospedale Regionale Lugano Ente Ospedaliero Cantonale (EOC), Via Tesserete 46, 6903 Lugano, Switzerland; 3 Private Practice, 503 Tak Shing House, 20 Des Voeux Road, Central, Hong Kong SAR, People’s Republic of China; 4 Discipline of Periodontology, Faculty of Dentistry, Prince Philip Dental Hospital, The University of Hong Kong, 34 Hospital Road, Sai Ying Pun, Hong Kong SAR, People’s Republic of China; 5 Center of Oral and Maxillofacial Surgery Studies, Faculty of Dentistry, Universiti Teknologi MARA Sungai Buloh Campus, Jalan Hospital, Selangor Darul Ehsan 47000 Sungai Buloh, Malaysia

**Keywords:** Aesthetics, Perception, Anthropometry, Facial proportion, Facial angle

## Abstract

**Background:**

The established recommendations and guidelines regarding ideal measurements for an attractive face are mostly based on data gathered among Caucasian population. The aim of this study was to examine the relationship between perception of 3-dimensional facial attractiveness and golden ratio, neoclassical canons, ‘ideal’ ratios and ‘ideal’ angles in Hong Kong Chinese.

**Methods:**

Thirty 3-D photographs (15 males and 15 females) were shown to 101 laypersons and 60 patients seeking orthognathic treatment. The photographs were rated based on a 100 mm visual analogue scale (VAS) from 0 (very unattractive) to 100 (very attractive).

**Results:**

More than half of the measurements (42/77) in females and thirty-two measurements in males were found to be significantly different from the ideal target value (*p* < 0.05) upon the comparison of the attractive faces with golden ratio, neoclassical canons, ‘ideal’ ratios and ‘ideal’ angles. Meanwhile, correlation tests between VAS scores and the parameters detected significant results (*p* < 0.05) in only six ratios, eight angles, one neoclassical canon and one proportion.

**Conclusions:**

Despite several renowned ‘ideal’ parameters of attractive faces that have been recommended in the literature, only a few of them were found to be significantly correlated with attractive faces in Hong Kong Chinese.

## Background

Face is known to be the key factor in the perception of physical attractiveness. Objective aesthetic criteria are important to evaluate and analyse patients who undergo aesthetic surgical procedures [1]. The computation of facial attractiveness has recently emerged as a new area of research. The groundwork, however, for the success of such technology relies on quantitative methods to define facial attractiveness [2]. Therefore, researchers have intended to quantify the perception of beauty using different facial parameters instead of subjective interpretations or individual observations of facial attractiveness [3].

Recommendations and guidelines in the literature regarding ideal measurements for an attractive face are mostly based on some recommended golden ratio, neoclassical canons, ‘ideal’ ratios and ‘ideal’ angles. Their ideal target values are normally based on average faces, faces perceived as ‘beautiful’ or authors’ preferences [4, 5]. Such ideal target values are presumably associated with attractive faces regardless of age, gender and ethnicity. Clinically, they are aimed as reference points by orthodontists, oral and maxillofacial surgeons and plastic surgeons for final treatment outcomes in both genders and all races. However, perception of facial attractiveness differs with race and ethnicity resulting in the application of some conventional rules for ideal facial attractiveness inappropriate [6].

Proportional or ratio analyses are considered to be more suitable in facial aesthetic appreciation as the comparison of absolute values among ethnicities and between genders is difficult due to inherent variation [7]. Up to date, many researchers [8–11] have suggested ‘ideal’ ratios or angles based on their collected data, which ultimately led to a plethora of ‘ideal’ parameters related to facial aesthetics available nowadays in literature.

The researchers replaced the traditional cephalometric analysis to evaluate facial attractiveness with photographs some time ago. Today, the emerging 3-dimensional (3-D) photographic technology provides a more vivid and realistic appreciation of facial aesthetics. Full facial landscapes can be acquired quickly and accurately in a noninvasive manner using 3-D imaging techniques [12].

It is important to respect and appreciate the underlying ethnic differences for the success of aesthetic surgery [1]. Therefore, the objective of this study is to examine the relationship between the perception of 3-dimensional facial attractiveness and golden ratio, neoclassical canons, ‘ideal’ ratios and ‘ideal’ angles in Hong Kong Chinese.

## Materials and methods

The ethical approval for this study was granted by the local authority (Institutional Review Board no.: UW 12–066). The study was conducted at Prince Philip Dental Hospital, The University of Hong Kong. Prior written informed consent was obtained from all the model volunteers and judges.

### 3-D photograph

Ninety Chinese dental students ranging in age from 20 to 27 years old (mean = 22.8) have volunteered and been recruited as 3-dimensional (3-D) photograph models in this study. These 90 volunteers consisted of 30 individuals presenting dento-skeletal classes 1, 2, and 3 each. Each model obtained a 3-dimensional photograph in a neutral facial expression using the *3dMDface* stereophotography system (3dMD, Atlanta, USA). To reduce potentially extraneous aesthetic factors during the photo-shooting session, their hair was covered. The make-up and jewellery were also removed.

**Fig. 1:**
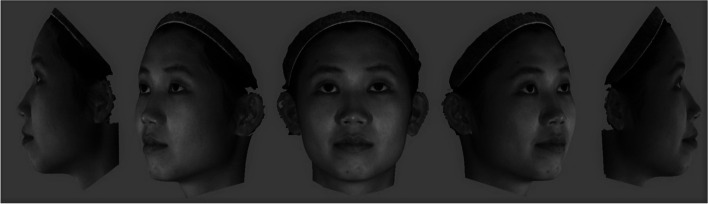
3D photographs of a female model from different angles adopted from the video sequence

The 3-D photos of the models were imported into the 3dMDVultus software (3dMD LLC, Atlanta, GA, USA). Five models each for three different dento-skeletal patterns (classes 1, 2, and 3) of both genders were then selected randomly using the random number generator function of Microsoft Excel (Microsoft Office Professional Plus 2016, Microsoft Corporation). This resulted in 15 male and 15 female 3-D photographs with diverse dental and skeletal patterns. A 10-s video was generated for each 3-D facial photograph rotating around its y-axis, starting from left to right face (Fig. 1). All videos were converted into black and white to reduce potential bias caused by skin colour and complexion.

### Judges

Judges for this study were recruited from the pool of consecutive patients attending the oral and maxillofacial discipline seeking orthognathic treatment, as well as from those patients who have attended the Reception and Primary Care Clinic of the same hospital for reasons other than potential orthognathic treatment. Only Hong Kong Chinese between 18 to 40 years old were included.

The judges were instructed to evaluate 30 videos based on a 100 mm visual analogue scale (VAS) from 0 (very unattractive) to 100 (very attractive). No time limit was set for the evaluation of the 3-D photographs. The consecutive videos were played only after the score for the previous video was recorded by the judges. The mean VAS score of each judging panel represented the final facial aesthetic score for each 3-D photograph.

**Fig. 2:**
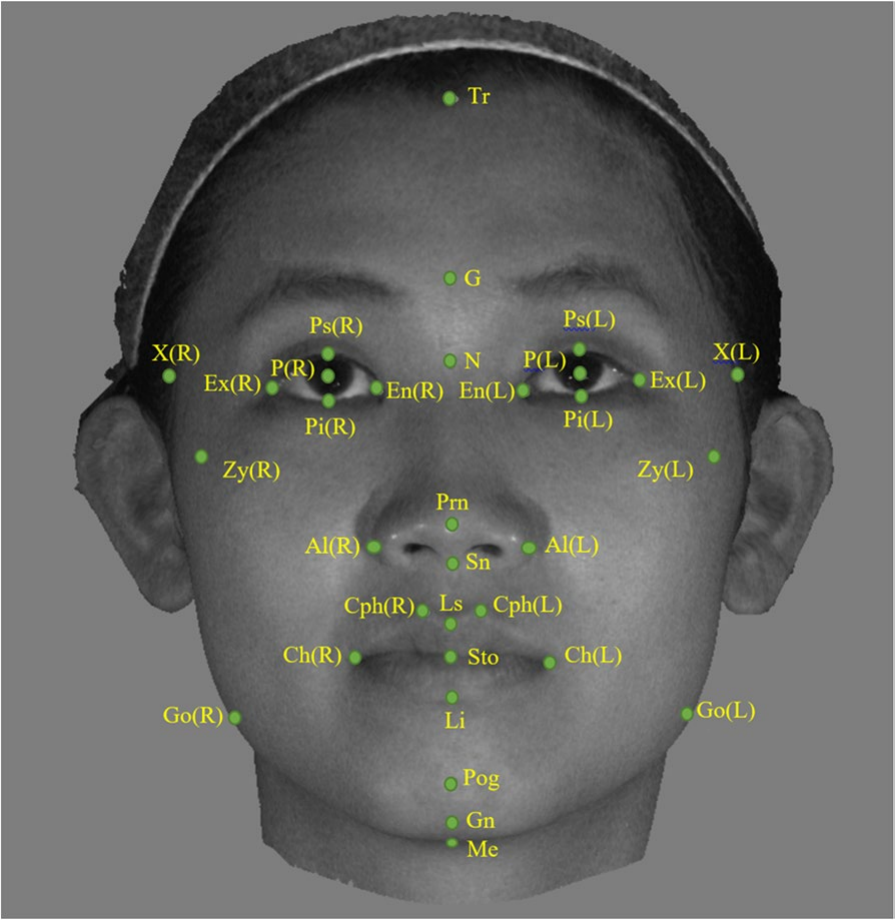
Soft tissue anthropometry landmarks

The soft tissue landmarks for each 3-D photograph were plotted using the 3dMDVultus software (3dMD LLC, Atlanta, GA, USA). The landmarks normally identified in the profile view of 2-D photographs were plotted in the midline of the frontal 3-D photograph view to prevent measurement errors due to horizontal deviations. The soft tissue landmarks used in this study are shown in Fig. 2.

**Fig. 3:**
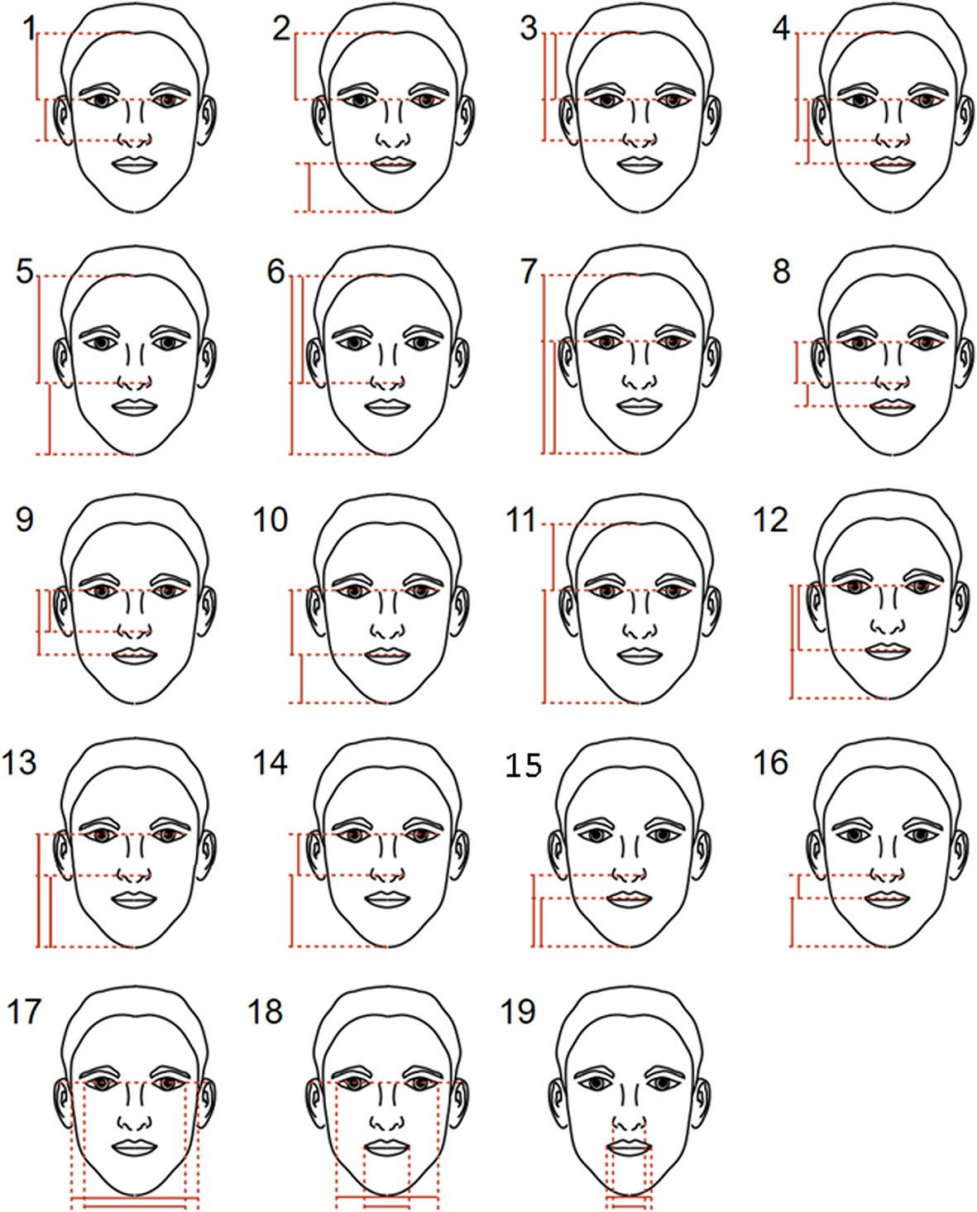
Golden ratio-related parameters measured in this study

**Fig. 4:**
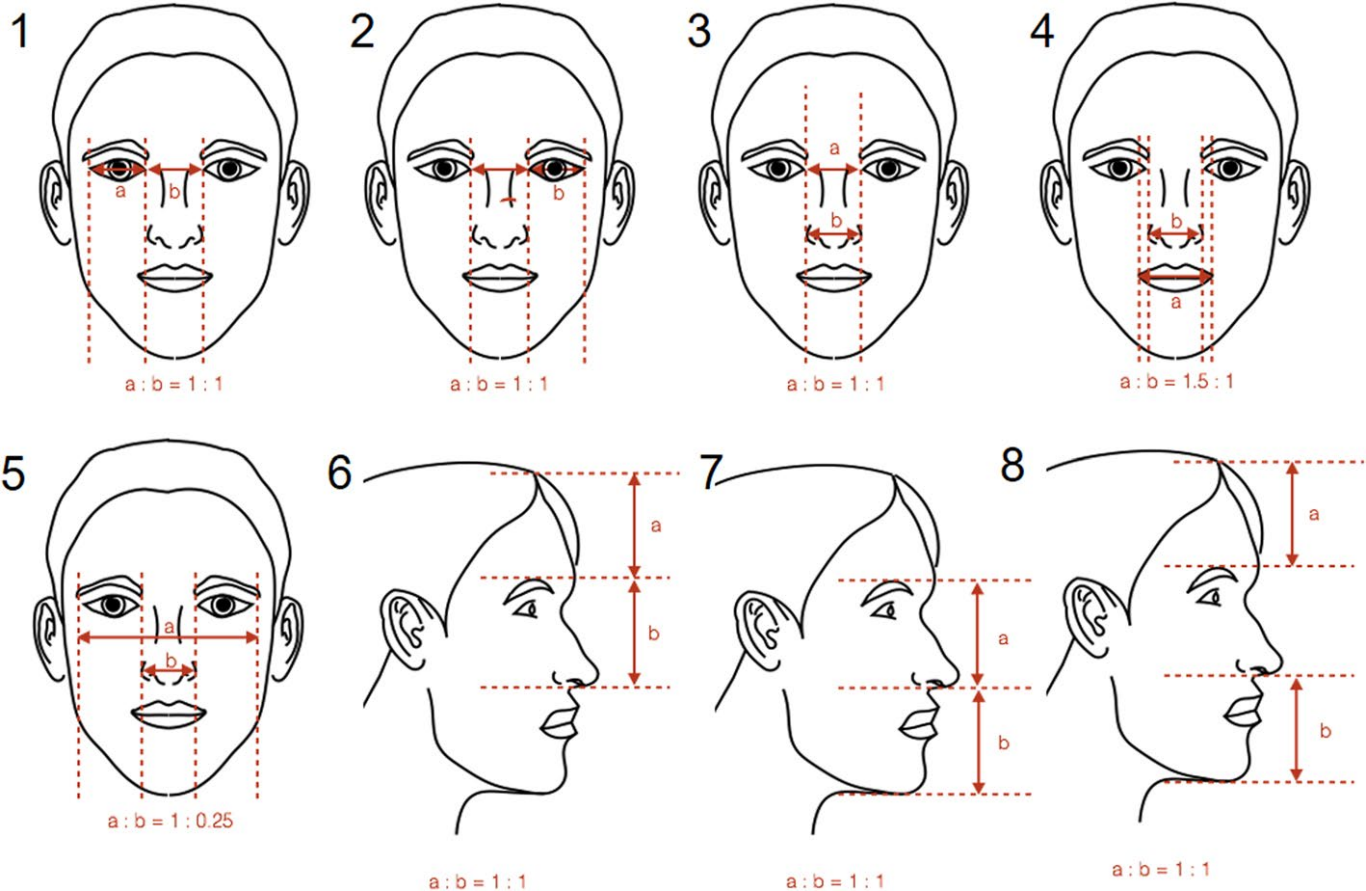
Neoclassical canons measured in this study

**Fig. 5:**
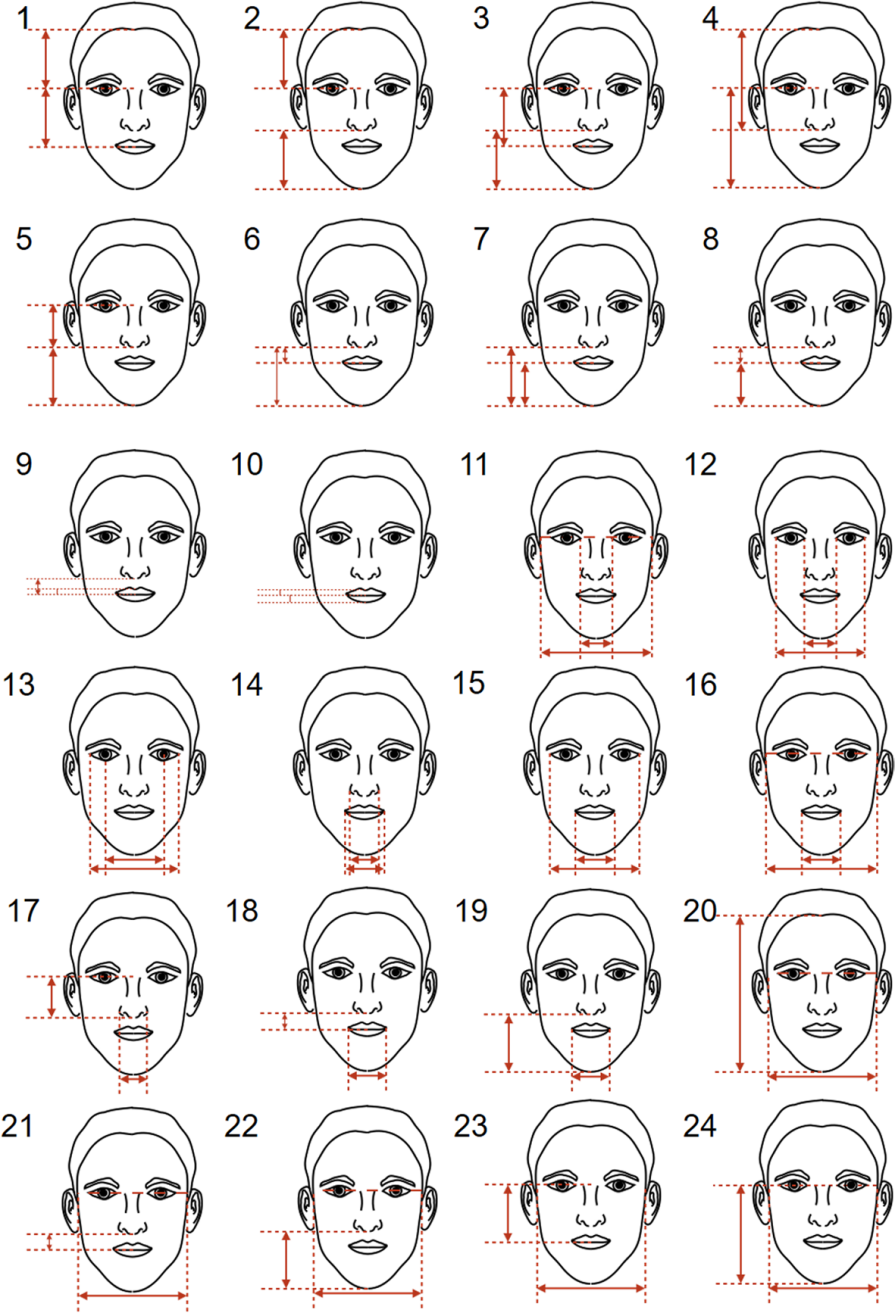
‘Ideal’ ratio parameters measured in this study

**Fig. 6:**
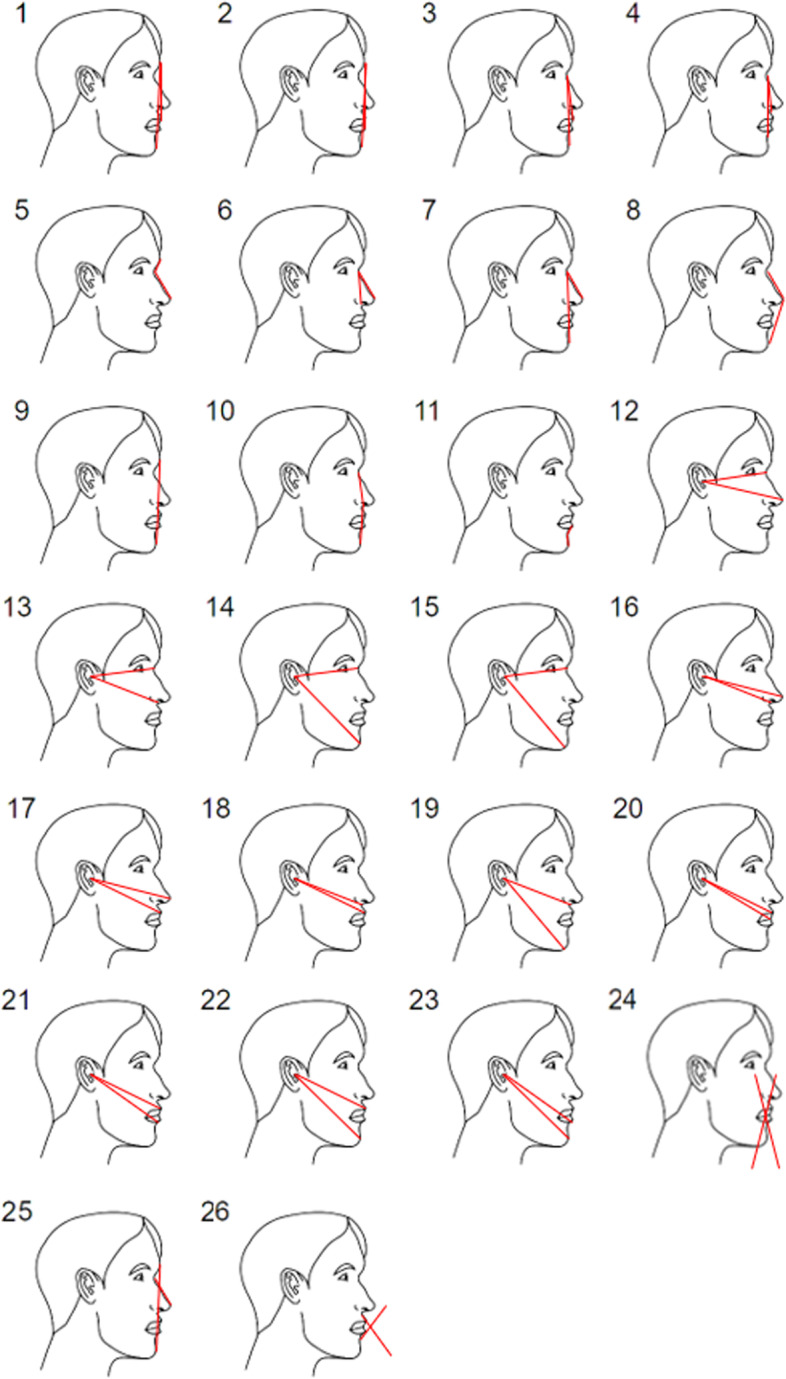
‘Ideal’ angle parameters measured in this study

According to already established methodologies [4, 5, 13–16], the golden ratio, neoclassical canons, ‘ideal’ ratios and ‘ideal’ angles were applied in this study (Figs. 3, 4, 5 and 6). The differences between the measured parameters and the ‘ideal’ reference values were calculated.

All 3-D photos were remeasured 2 weeks after the first measurement, and the technical error measurement (TEM) was calculated with the Dahlberg formula as follows [17]:

$$\mathrm{D }=\sqrt{\sum_{i=1}^{N}\frac{{d}_{i}^{2}}{2N}}$$

where ${d}_{i}$ is the difference between the first and the second measurements and *N* is the sample size that was remeasured. Subsequently, the relative TEM (% TEM) was calculated as follows:

$$\%\;\mathrm{TEM}=\frac{\mathrm{TEM}}{\overline{\mathrm x}}\times100\%$$

where $\overline{\mathrm{x}}$ is the sample mean. The acceptable range for intra-examiner % TEM is < 1.5% [18].

### Statistical analysis

Descriptive statistics were used to analyse the demographic parameters of the judges. Paired *t*-test was performed to examine any differences between the mean VAS scores of orthognathic versus non-orthognathic judges.

One-sample Wilcoxon signed-rank test was used to examine to differences between attractive faces with the ideal value of recommended parameters. Five female and male models with the highest VAS scores were selected for this analysis. Subsequently, the Pearson correlations test was used to examine the association between the mean VAS scores with all the measured independent variables.

A *p*-value of < 0.05 was considered significant for all statistical tests. All data unless specified were analysed using the SPSS Statistics software version 23.0 (Armonk, NY: IBM Corp, USA).

## Results

A total of 180 patients were recruited consecutively to be judges for this study; however, incomplete data was found in 17 of them. As a result, 163 judges (age: 27.1 ± 6.1 years old) were analysed for this study. Among them, 25 of 62 orthognathic and 40 of 101 layperson judges were male. All intra-assessor’s measured landmarks % TEMs were within the acceptable range (0.12–1.23%).

While the recorded raw VAS scores ranged from 0 to 99.5, the mean VAS scores for the 3-D photographs were 49.76 ± 6.14 for male and 47.97 ± 7.62 for female 3-D photographs. No significant difference (*p* = 0.161) existed for the mean VAS score (− 0.67 ± 2.57) between orthognathic and layperson judges for all 3-D photos.

### Attractive faces versus recommended parameters

More than half of the measurements (42/77) were found to be significantly different from the ideal target value (*p* < 0.05) upon comparison of the attractive female faces with golden ratio (Table 1), neoclassical canons (Table 2), ‘ideal’ ratios (Table 3) and ‘ideal’ angles (Table 4). An attractive female face can be interpreted as having a shorter (n-sn) and broader (al-al) nose, broader inter-endocanthus and inter-exocanthus width, shorter lower facial third, thicker vermillion of both upper and lower lips, shorter upper lip, flatter labio-mental fold and retrusive mandible comparing to the recommended ideal faces by reading these results together. Majority of these findings were found significant in both orthognathic and layperson judges.

**Table 1:** Comparison of attractive male and female faces with golden ratio (1.618)

Parameters	Descriptions	Attractive male faces (*n* = 5)						Attractive female faces (*n* = 5)					
		Total judges		Layman judges		Orthognathic judges		Total judges		Layman judges		Orthognathic judges	
		Median	*p*-value	Median	*p*-value	Median	*p*-value	Median	*p*-value	Median	*p*-value	Median	*p*-value
G1	Tr-Ex′: Ex′-Al′	1.78	0.50	1.78	0.14	1.78	0.50	1.88	0.04*	1.88	0.04*	1.89	0.04*
G2	Tr-Ex′: Ch′-Me	1.79	0.89	1.85	0.35	1.79	0.89	1.75	0.35	1.75	0.89	1.56	0.35
G3	Tr-Al′: Tr-Ex′	1.56	0.69	1.56	0.23	1.56	0.69	1.52	0.04*	1.52	0.04	1.52	0.04*
G4	TR-Al′: Ex′-Ch′	1.75	0.35	1.75	0.14	1.75	0.35	1.7	0.04*	1.7	0.04*	1.66	0.04*
G5	Tr-Al′: Al′-Me	1.51	0.50	1.55	0.89	1.51	0.50	1.53	0.08	1.53	0.04*	1.52	0.08
G6	Tr-Me: Tr-Al′	1.56	0.23	1.51	0.08	1.56	0.23	1.55	0.04*	1.55	0.04*	1.57	0.04*
G7	Tr-Me: Ex′-Me	1.67	0.89	1.67	0.35	1.67	0.89	1.7	0.04*	1.7	0.04*	1.66	0.04*
G8	Ex′-Al′: Al-Ch′	1.4	0.14	1.34	0.14	1.4	0.14	1.29	0.04*	1.29	0.04*	1.22	0.04*
G9	Ex′-Ch′: Ex′-Al′	1.53	0.50	1.65	0.89	1.53	0.50	1.68	0.35	1.68	0.23	1.69	0.35
G10	Ex′-Ch′: Ch′-Me	1.44	0.14	1.53	0.50	1.44	0.14	1.64	0.69	1.64	0.23	1.42	0.69
G11	Me-Ex′: Ex′-Tr	1.4	0.35	1.4	0.23	1.4	0.35	1.38	0.04*	1.38	0.04*	1.45	0.04*
G12	Ex′-Me: Ex′-Ch′	1.63	0.89	1.63	0.69	1.63	0.89	1.57	0.34	1.57	0.50	1.59	0.34
G13	Ex′-Me: Al′-Me	1.41	0.04*	1.41	0.04*	1.41	0.04*	1.42	0.04*	1.42	0.04*	1.41	0.04*
G14	Al′-Me: Ex′-Al′	1.81	0.23	1.95	0.23	1.81	0.23	1.87	0.04*	1.87	0.04*	1.89	0.04*
G15	Al′-Me: Ch′-Me	1.71	0.14	1.74	0.08	1.71	0.14	1.78	0.23	1.78	0.35	1.7	0.23
G16	Ch′-Me: Al′-Ch′	1.41	0.08	1.34	0.08	1.41	0.08	1.28	0.35	1.28	0.35	1.37	0.35
G17	X(R)-X(L): Ex(R)-Ex(L)	1.58	0.22	1.58	0.35	1.58	0.22	1.53	0.04*	1.53	0.08	1.56	0.04*
G18	Ex(R)-Ex(L): Ch(R)-Ch(L)	1.96	0.04*	1.94	0.04	1.96	0.04*	1.87	0.04*	1.87	0.04*	1.87	0.04*
G19	Ch(R)-Ch(L): Al(R)-Al(L)	1.26	0.04*	1.39	0.04	1.26	0.04*	1.39	0.04*	1.39	0.04*	1.39	0.04*

^*^Significant *p*-value < 0.05

**Table 2:** Comparison of attractive male and female faces with neoclassical canons

Parameters	Descriptions	Ideal target value	Attractive male faces (*n* = 5)						Attractive female faces (*n* = 5)					
			Total judges		Layman judges		Orthognathic judges		Total judges		Layman judges		Orthognathic judges	
			Median	*p*-value	Median	*p*-value	Median	*p*-value	Median	*p*-value	Median	*p*-value	Median	*p*-value
C1	Orbital canon (right) Ex(R)-En(R): Ex(R)-Ex(L)	1	1.78	0.50	1.78	0.14	1.78	0.50	1.88	0.04*	1.88	0.04*	1.89	0.04*
C2	Orbital canon (left) Ex(L)-En(L): Ex(R)-Ex(L)	1	1.79	0.89	1.85	0.35	1.79	0.89	1.75	0.35	1.75	0.89	1.56	0.35
C3	Orbito-nasal canon En(R)-En(L): Al(R)-Al(L)	1	1.56	0.69	1.56	0.23	1.56	0.69	1.52	0.04*	1.52	0.04	1.52	0.04*
C4	Naso-oral canon Ch(R)-Ch(L): Al(R)-Al(L)	1.5	1.75	0.35	1.75	0.14	1.75	0.35	1.7	0.04*	1.7	0.04*	1.66	0.04*
C5	Naso-facial canon Al(R)-Al(L): Zy(R)-Zy(L)	0.25	1.51	0.50	1.55	0.89	1.51	0.50	1.53	0.08	1.53	0.04*	1.52	0.08
C6	Three section facial profile canon (upper vs middle face) Tr-N: N-Sn	1	1.56	0.23	1.51	0.08	1.56	0.23	1.55	0.04*	1.55	0.04*	1.57	0.04*
C7	Three section facial profile canon (middle vs lower face) N-Sn: Sn-Gn	1	1.67	0.89	1.67	0.35	1.67	0.89	1.7	0.04*	1.7	0.04*	1.66	0.04*

^*^Significant *p*-value < 0.05

**Table 3:** Comparison of attractive male and female faces with ‘ideal’ ratios

Parameters	Descriptions	Ideal target value	Attractive male faces (*n* = 5)						Attractive female faces (*n* = 5)					
			Total judges		Layman judges		Orthognathic judges		Total judges		Layman judges		Orthognathic judges	
			Median	*p*-value	Median	*p*-value	Median	*p*-value	Median	*p*-value	Median	*p*-value	Median	*p*-value
R1	Tr-N: N-Sto	1	0.86	0.14	0.86	0.22	0.86	0.14	1.00	0.72	1.00	0.72	0.96	0.50
R2	Tr-N: Sn-Me	1	0.94	0.69	0.94	0.69	0.94	0.69	1.10	0.23	1.10	0.23	0.97	0.68
R3	N-Sto: Sn-Me	1	1.07	0.04*	1.07	0.04*	1.07	0.04*	1.12	0.04*	1.12	0.04*	1.07	0.04*
R4	Tr-Sn: N-Me	1	0.99	1.00	0.99	0.50	0.99	1.00	1.09	0.07	1.09	0.07	1.02	0.07
R5	N-Sn: Sn-Me	0.754	0.77	0.23	0.77	0.23	0.77	0.23	0.80	0.14	0.80	0.14	0.79	0.89
R6	Sn-Sto: Sn-Me	0.333	0.30	0.22	0.30	0.34	0.30	0.22	0.32	0.34	0.32	0.34	0.32	0.89
R7	Sto-Me: Sn-Me	0.667	0.70	0.14	0.70	0.14	0.70	0.14	0.70	0.14	0.70	0.14	0.69	0.23
R8	Sn-Sto: Sto-Me	0.5	0.43	0.23	0.43	0.35	0.43	0.23	0.45	0.35	0.45	0.35	0.47	0.50
R9	Ls-Sto: Sn-Sto	0.36	0.40	0.04*	0.40	0.06	0.40	0.04*	0.47	0.04*	0.47	0.04*	0.40	0.04*
R10	Ls-Sto: Sto-Li	0.88	1.12	0.72	1.12	0.23	1.12	0.72	1.01	0.50	1.01	0.50	1.13	0.35
R11	En(R)-En(L): X(R)-X(L)	0.2	0.2	0.04*	0.25	0.04*	0.2	0.04*	0.27	0.04*	0.27	0.04*	0.26	0.04*
R12	En(R)-En(L): Ex(R)-Ex(L)	0.333	0.38	0.04*	0.38	0.04*	0.38	0.04*	0.40	0.04*	0.40	0.04*	0.40	0.04*
R13	P(R)-P(L): Ex(R)-Ex(L)	0.7	0.65	0.04*	0.65	0.04*	0.65	0.04*	0.69	0.20	0.69	0.20	0.69	0.20
R14	Al(R)-Al(L): Ch(R)-Ch(L)	0.625	0.79	0.04*	0.79	0.04*	0.79	0.04*	0.72	0.04*	0.72	0.04*	0.72	0.04*
R15	Ch(R)-Ch(L): Ex(R)-Ex(L)	0.6	0.51	0.04*	0.51	0.04*	0.51	0.04*	0.53	0.04*	0.53	0.04*	0.53	0.04*
R16	Ch(R)-Ch(L): X(R)-X(L)	0.4	0.32	0.04*	0.32	0.04*	0.32	0.04*	0.36	0.04*	0.36	0.04*	0.36	0.04*
R17	Al(R)-Al(L): N-Sn	0.625	0.64	0.35	0.64	0.69	0.64	0.35	0.68	0.04*	0.68	0.04*	0.73	0.04*
R18	Sn-Sto: Ch(R)-Ch(L)	0.4	0.48	0.22	0.48	0.23	0.48	0.22	0.42	0.10	0.42	0.10	0.43	0.08
R19	Sn-Me: Ch(R)-Ch(L)	1.33	1.47	0.08	1.47	0.10	1.47	0.08	1.33	1.00	1.33	1.00	1.40	0.50
R20	X(R)-X(L): Tr-Me	0.783	0.80	0.50	0.80	0.50	0.80	0.50	0.77	0.69	0.77	0.69	0.79	0.50
R21	Sn-Sto: X(R)-X(L)	0.225	0.14	0.04*	0.14	0.04*	0.14	0.04*	0.14	0.04*	0.14	0.04*	0.15	0.04*
R22	Sn-Me: X(R)-X(L)	0.53	0.48	0.04*	0.48	0.10	0.48	0.04*	0.47	0.04*	0.47	0.04*	0.47	0.04*
R23	N-Sto: X(R)-X(L)	0.535	0.52	0.04*	0.52	0.50	0.52	0.04*	0.52	0.10	0.52	0.10	0.52	0.10
R24	N-Me: X(R)-X(L)	0.86	0.82	0.04*	0.82	0.50	0.82	0.04*	0.83	0.07	0.83	0.07	0.83	0.07

^*^Significant *p*-value < 0.05

**Table 4:** Comparison of attractive male and female faces with ‘ideal’ angles

Parameters	Descriptions	Ideal target value	Attractive male faces (*n* = 5)						Attractive female faces (*n* = 5)					
			Total judges		Layman judges		Orthognathic judges		Total judges		Layman judges		Orthognathic judges	
			Median	*p*-value	Median	*p*-value	Median	*p*-value	Median	*p*-value	Median	*p*-value	Median	*p*-value
A1	Lsp-G-Pog	6.3	6.0	0.89	6.0	0.50	6.0	0.89	5.5	0.69	5.5	0.69	6.2	0.89
A2	Lip-G-Pog	3.3	3.6	0.50	2.9	0.69	3.6	0.50	3.2	0.69	3.2	0.69	5.3	0.23
A3	Lsp-N-Pog	5.9	7.4	0.14	7.4	0.23	7.4	0.14	7.9	0.08	7.9	0.08	8.4	0.04
A4	A-N-B	7.1	8.9	0.23	7.0	0.89	8.9	0.23	6.2	0.79	6.2	0.79	6.2	0.79
A5	G-N-Pn	140.3	140.8	0.69	140.8	0.89	140.8	0.69	145.9	0.08	145.9	0.08	147.2	0.04
A6	Pn-N-Sn	22.5	19.5	0.04	19.5	0.50	19.5	0.04	18.3	0.04	18.3	0.04	18.3	0.04
A7	Pn-N-Pog	27.5	30.6	0.14	30.6	0.35	30.6	0.14	26.4	0.35	26.4	0.35	27.2	0.89
A8	N-Pn-Pog	129.5	131.1	0.69	133.3	0.89	131.1	0.69	138.2	0.08	138.2	0.08	134.6	0.08
A9	G-Sn-Pog	170	163.5	0.04	164.4	0.08	163.5	0.04	167.2	0.35	167.2	0.35	167.2	0.04
A10	N-Sn-Pog	163	161.5	0.04	162.4	0.50	161.5	0.04	160.8	0.89	160.8	0.89	160.8	0.68
A11	Lip-B-Pog	125.5	156.6	0.04	156.4	0.04	156.6	0.04	162.4	0.04	162.4	0.04	155.1	0.04
A12	N-Po-Pn	23.6	19.8	0.04	19.7	0.04	19.8	0.04	20.4	0.04	20.4	0.04	19.5	0.04
A13	N-Po-Sn	28.5	24.8	0.04	25.0	0.04	24.8	0.04	25.2	0.04	25.2	0.04	25.1	0.04
A14	N-Po-Pog	54.4	47.3	0.04	47.3	0.04	47.3	0.04	46.6	0.04	46.6	0.04	46.4	0.04
A15	N-Po-Gn	57	51.5	0.04	51.5	0.08	51.5	0.04	51.5	0.04	51.5	0.04	51.1	0.04
A16	Pn-Po-Sn	7	7.1	0.69	7.1	0.50	7.1	0.69	7.4	0.69	7.4	0.69	7.0	1
A17	Pn-Po-Ls	14.5	11.7	0.08	12.4	0.69	11.7	0.08	11.9	0.04	11.9	0.04	12.0	0.04
A18	Sn-Po-Ls	7	5.0	0.08	6.6	0.23	5.0	0.08	5.2	0.04	5.2	0.04	5.5	0.08
A19	Sn-Po-Gn	36.5	27.5	0.04	27.5	0.04	27.5	0.04	26.3	0.04	26.3	0.04	26.5	0.04
A20	Ls-Po-Sto	2.8	3.6	0.04	3.3	0.04	3.6	0.04	4.4	0.04	4.4	0.04	4.3	0.04
A21	Ls-Po-Li	7.1	7.7	0.69	7.5	0.69	7.7	0.69	8.1	0.04	8.1	0.04	8.1	0.14
A22	Ls-Po-Pog	17.1	16.9	0.69	16.7	0.23	16.9	0.69	16.6	0.35	16.6	0.35	16.6	0.35
A23	Li-Po-Pog	12.5	9.9	0.04	9.5	0.04	9.9	0.04	8.1	0.04	8.1	0.04	8.7	0.04
A24	(Sn-Lsp)-(Pog-Lip)	157.3	168.5	0.14	168.5	0.23	168.5	0.14	154.0	0.35	154.0	0.35	154.0	0.35
A25	(G-Pog)-(N-Pn)	35	31.0	0.14	27.8	0.07	31.0	0.14	29.0	0.04	29.0	0.04	29.0	0.04
A26	(B-Lip)-(Lsp-A)	125	148.0	0.04	148.0	0.04	148.0	0.04	142.0	0.14	142.0	0.14	139.0	0.23

^*^Significant *p*-value < 0.05

On the other hand, 32 measurements were found to be statistically significant in the measured parameters for male faces (Tables 1, 2, 3 and 4). A shorter and broader nose, broader inter-endocanthus and inter-exocanthus width, shorter lower facial third, retrusive mandible, thicker vermillion of upper lip, shorter upper lip and flatter labio-mental fold than the recommended value were found to be more attractive in male faces based on the analyses. Again, majority of the significant findings were found in both groups of judges.

### Analyses based on VAS scores

Correlation tests between VAS scores and the parameters detected significant results in six ratios, eight angles, one neoclassical canon and one proportion (Table 5). These significant results were recorded at ratios 3, 5, 13, 17, 19 and 22, in angles 4, 7, 9, 10, 14, 15, 19, 22 and 23, in neoclassical canon 5, as well as to proportion 14. In subgroup analyses, it became obvious that significant correlations within the overall analysis of all models turned out not to be always significant for analyses of female or male faces and vice versa. The same applied to the results of the overall judges’ analyses when compared with the subgroup results of orthognathic and layperson judges respectively.

**Table 5:** The correlations between the mean VAS scores and the deviations of the measured parameters from ideal target values

Parameters	All models						Female models						Male models					
	Overall		Orthognathic judges		Layperson judges		Overall		Orthognathic judges		Layperson judges		Overall		Orthognathic judges		Layperson judges	
	*r*	*p*	*r*	*p*	*r*	*p*	*r*	*p*	*r*	*p*	*r*	*p*	*r*	*p*	*r*	*p*	*r*	*p*
R1	− 0.073	0.71	− 0.136	0.48	− 0.031	0.87	− 0.116	0.68	− 0.139	0.62	− 0.099	0.73	0.077	0.79	− 0.014	0.96	0.132	0.65
R2	0.089	0.65	0.041	0.83	0.116	0.55	0.051	0.86	0.053	0.85	0.048	0.87	0.217	0.46	0.129	0.66	0.267	0.36
R3	0.379	0.04*	0.396	0.03	0.358	0.06	0.336	0.22	0.387	0.15	0.298	0.28	0.423	0.13	0.391	0.17	0.432	0.12
R4	0.109	0.57	0.063	0.75	0.135	0.49	0.040	0.89	0.049	0.86	0.034	0.90	0.276	0.34	0.187	0.52	0.324	0.26
R5	0.413	0.03*	0.414	0.03*	0.403	0.03*	0.274	0.32	0.289	0.30	0.259	0.35	0.570	0.03*	0.541	0.046*	0.574	0.03*
R6	− 0.001	1.00	0.061	0.75	− 0.038	0.84	0.193	0.49	0.311	0.26	0.117	0.68	− 0.231	0.43	− 0.227	0.43	− 0.228	0.43
R7	− 0.159	0.41	− 0.225	0.24	− 0.115	0.55	− 0.256	0.36	− 0.336	0.22	− 0.202	0.47	0.030	0.92	− 0.013	0.97	0.056	0.85
R8	0.083	0.67	0.147	0.45	0.042	0.83	0.247	0.37	0.355	0.19	0.177	0.53	− 0.137	0.64	− 0.124	0.67	− 0.142	0.63
R9	− 0.039	0.84	− 0.138	0.47	0.023	0.91	− 0.017	0.95	− 0.096	0.73	0.031	0.91	− 0.031	0.92	− 0.148	0.61	0.043	0.89
R10	0.120	0.54	0.100	0.61	0.129	0.50	0.113	0.69	0.145	0.61	0.091	0.75	0.106	0.72	0.034	0.91	0.149	0.61
R11	0.014	0.94	0.044	0.82	− 0.004	0.98	0.437	0.10	0.474	0.07	0.405	0.13	− 0.479	0.08	− 0.447	0.11	− 0.487	0.08
R12	0.129	0.51	0.158	0.41	0.108	0.58	0.482	0.07	0.513	0.05	0.452	0.09	− 0.510	0.06	− 0.469	0.09	− 0.523	0.06
R13	0.375	0.05*	0.307	0.11	0.408	0.03*	0.440	0.10	0.388	0.15	0.461	0.08	0.346	0.23	0.274	0.34	0.382	0.18
R14	0.265	0.16	0.309	0.10	0.232	0.23	0.276	0.32	0.320	0.25	0.243	0.38	0.180	0.54	0.192	0.51	0.169	0.56
R15	0.241	0.21	0.229	0.23	0.242	0.21	0.419	0.12	0.386	0.16	0.428	0.11	0.102	0.73	0.181	0.54	0.050	0.87
R16	0.167	0.39	0.168	0.38	0.163	0.40	0.490	0.06	0.474	0.07	0.488	0.07	− 0.135	0.65	− 0.071	0.81	− 0.171	0.56
R17	0.294	0.12	0.357	0.06	0.248	0.19	0.526	0.04*	0.590	0.02*	0.475	0.07	− 0.298	0.30	− 0.211	0.47	− 0.345	0.23
R18	− 0.352	0.06	− 0.328	0.08	− 0.358	0.06	− 0.455	0.09	− 0.415	0.12	− 0.468	0.08	− 0.318	0.27	− 0.326	0.26	− 0.305	0.29
R19	− 0.455	0.01*	− 0.488	0.01*	− 0.424	0.02*	− 0.644	0.01*	− 0.707	> 0.01*	− 0.592	0.02*	− 0.234	0.42	− 0.260	0.37	− 0.213	0.47
R20	0.270	0.16	0.375	0.045*	0.198	0.30	0.348	0.20	0.454	0.09	0.276	0.32	0.085	0.77	0.178	0.54	0.025	0.93
R21	− 0.286	0.13	− 0.264	0.17	− 0.293	0.12	− 0.258	0.35	− 0.217	0.44	− 0.277	0.32	− 0.362	0.20	− 0.345	0.23	− 0.364	0.20
R22	− 0.383	0.04*	− 0.428	0.02*	− 0.345	0.07	− 0.388	0.15	− 0.472	0.08	− 0.329	0.23	− 0.355	0.21	− 0.339	0.24	− 0.356	0.21
R23	− 0.117	0.55	− 0.148	0.45	− 0.095	0.63	− 0.229	0.41	− 0.292	0.29	− 0.186	0.51	0.009	0.98	0.006	0.99	0.011	0.97
R24	− 0.307	0.11	− 0.365	0.05	− 0.264	0.17	− 0.343	0.21	− 0.447	0.10	− 0.272	0.33	− 0.243	0.40	− 0.229	0.43	− 0.247	0.40
A1	− 0.048	0.80	− 0.053	0.79	− 0.044	0.82	− 0.203	0.47	− 0.190	0.50	− 0.207	0.46	0.215	0.46	0.197	0.50	0.221	0.45
A2	− 0.279	0.14	− 0.296	0.12	− 0.262	0.17	− 0.313	0.26	− 0.328	0.23	− 0.297	0.28	− 0.249	0.39	− 0.258	0.37	− 0.237	0.41
A3	− 0.061	0.75	− 0.084	0.66	− 0.045	0.82	− 0.221	0.43	− 0.221	0.43	− 0.216	0.44	0.161	0.58	0.113	0.70	0.188	0.52
A4	0.523	< 0.01*	0.573	< 0.01*	0.480	0.01*	0.257	0.36	0.358	0.19	0.190	0.50	0.803	< 0.01*	0.785	< 0.01*	0.795	< 0.01*
A5	0.057	0.77	0.069	0.72	0.048	0.80	0.105	0.71	0.130	0.65	0.088	0.76	0.209	0.47	0.274	0.34	0.163	0.58
A6	0.097	0.62	0.139	0.47	0.068	0.72	0.248	0.37	0.253	0.36	0.240	0.39	− 0.277	0.34	− 0.183	0.53	− 0.329	0.25
A7	0.202	0.29	0.257	0.18	0.163	0.40	0.044	0.88	0.098	0.73	0.011	0.97	0.328	0.25	0.365	0.20	0.298	0.30
A8	− 0.308	0.10	− 0.357	0.06	− 0.270	0.16	− 0.126	0.66	− 0.170	0.54	− 0.096	0.73	− 0.464	0.10	− 0.496	0.07	− 0.433	0.12
A9	− 0.226	0.24	− 0.282	0.14	− 0.186	0.33	0.090	0.75	0.011	0.97	0.135	0.63	− 0.653	0.01*	− 0.680	0.01*	− 0.621	0.02*
A10	− 0.229	0.23	− 0.262	0.17	− 0.203	0.29	0.119	0.67	0.061	0.83	0.151	0.59	− 0.598	0.02*	− 0.587	0.03*	− 0.590	0.03*
A11	− 0.213	0.27	− 0.211	0.27	− 0.208	0.28	− 0.145	0.61	− 0.164	0.56	− 0.130	0.64	− 0.458	0.10	− 0.426	0.13	− 0.467	0.09
A12	− 0.025	0.90	− 0.064	0.74	0.001	1.00	− 0.179	0.52	− 0.235	0.40	− 0.142	0.61	0.336	0.24	0.326	0.26	0.335	0.24
A13	0.014	0.94	− 0.035	0.86	0.045	0.82	− 0.174	0.54	− 0.234	0.40	− 0.134	0.63	0.517	0.06	0.489	0.08	0.522	0.06
A14	− 0.389	0.04*	− 0.402	0.03*	− 0.372	0.047*	− 0.571	0.03*	− 0.613	0.02*	− 0.532^*^	0.04*	0.005	0.99	0.059	0.84	− 0.029	0.92
A15	− 0.371^*^	0.05	− 0.390^*^	0.04	− 0.350	0.06	− 0.549	0.03*	− 0.582	0.02*	− 0.516^*^	0.049*	0.001	1.00	0.016	0.96	− 0.009	0.98
A16	0.091	0.64	0.083	0.67	0.093	0.63	0.013	0.96	− 0.006	0.98	0.024	0.93	0.241	0.41	0.248	0.39	0.231	0.43
A17	0.047	0.81	0.150	0.44	-0.018	0.93	-0.052	0.86	0.051	0.86	-0.112	0.69	0.122	0.68	0.223	0.44	0.057	0.85
A18	− 0.120	0.54	− 0.008	0.97	− 0.185	0.34	− 0.154	0.58	− 0.053	0.85	− 0.210	0.45	− 0.106	0.72	0.016	0.96	− 0.179	0.54
A19	− 0.433	0.02*	− 0.414	0.03*	− 0.434	0.02*	− 0.580	0.02*	− 0.561	0.03*	− 0.578^*^	0.02*	− 0.215	0.46	− 0.192	0.51	− 0.224	0.44
A20	− 0.191	0.32	− 0.250	0.19	− 0.151	0.44	− 0.134	0.63	− 0.163	0.56	− 0.114	0.69	− 0.207	0.48	− 0.283	0.33	− 0.155	0.60
A21	− 0.166	0.39	− 0.224	0.24	− 0.127	0.51	− 0.115	0.68	− 0.154	0.58	− 0.089	0.75	− 0.157	0.59	− 0.213	0.47	− 0.118	0.69
A22	− 0.450	0.01*	− 0.489	0.01*	− 0.415	0.03*	− 0.596	0.02*	− 0.645	0.01*	− 0.553^*^	0.03*	− 0.239	0.41	− 0.257	0.38	− 0.222	0.45
A23	− 0.488	0.01*	− 0.465	0.01*	− 0.491	0.01*	− 0.645	0.01*	− 0.664	0.01*	− 0.619^*^	0.01*	− 0.252	0.39	− 0.177	0.55	− 0.292	0.31
A24	0.284	0.14	0.321	0.09	0.255	0.18	0.128	0.65	0.145	0.61	0.114	0.69	0.416	0.14	0.459	0.10	0.380	0.18
A25	0.080	0.68	0.135	0.48	0.044	0.82	− 0.037	0.90	0.014	0.96	− 0.067	0.81	0.232	0.42	0.259	0.37	0.210	0.47
A26	0.051	0.79	0.083	0.67	0.031	0.88	− 0.106	0.71	− 0.102	0.72	− 0.106	0.71	0.291	0.31	0.350	0.22	0.247	0.39
C1	− 0.161	0.41	− 0.176	0.36	− 0.147	0.45	− 0.463	0.08	− 0.483	0.07	− 0.439	0.10	0.404	0.15	0.374	0.19	0.413	0.14
C2	− 0.121	0.53	− 0.145	0.46	− 0.104	0.59	− 0.459	0.09	− 0.482	0.07	− 0.434	0.11	0.512	0.06	0.482	0.08	0.518	0.06
C3	0.266	0.16	0.280	0.14	0.251	0.19	0.135	0.63	0.132	0.64	0.134	0.64	0.468	0.09	0.500	0.07	0.436	0.12
C4	− 0.289	0.13	− 0.333	0.08	− 0.255	0.18	− 0.338	0.22	− 0.386	0.16	− 0.302	0.27	− 0.137	0.64	− 0.144	0.62	− 0.130	0.66
C5	0.410	0.03*	0.444	0.02*	0.380	0.04*	0.492	0.06	0.526	0.04*	0.461	0.08	0.257	0.38	0.291	0.31	0.230	0.43
C6	0.130	0.50	0.182	0.35	0.095	0.62	0.149	0.60	0.167	0.55	0.134	0.63	0.077	0.79	0.146	0.62	0.033	0.91
C7	− 0.061	0.75	− 0.014	0.94	− 0.089	0.65	− 0.048	0.86	− 0.048	0.86	− 0.047	0.87	− 0.136	0.64	− 0.061	0.84	− 0.180	0.54
C8	0.006	0.98	0.056	0.77	− 0.025	0.90	0.000	1.00	0.005	0.99	− 0.002	0.99	− 0.048	0.87	0.025	0.93	− 0.092	0.76
G1	− 0.163	0.40	− 0.152	0.43	− 0.166	0.39	− 0.179	0.52	− 0.140	0.62	− 0.199	0.48	− 0.068	0.82	− 0.055	0.85	− 0.075	0.80
G2	0.184	0.34	0.172	0.37	0.187	0.33	0.144	0.61	0.171	0.54	0.124	0.66	0.281	0.33	0.235	0.42	0.302	0.29
G3	0.168	0.38	0.162	0.40	0.167	0.39	0.120	0.67	0.083	0.77	0.139	0.62	0.149	0.61	0.137	0.64	0.153	0.60
G4	0.072	0.71	0.054	0.78	0.081	0.68	− 0.025	0.93	− 0.032	0.91	− 0.021	0.94	0.245	0.40	0.226	0.44	0.251	0.39
G5	0.207	0.28	0.162	0.40	0.230	0.23	0.137	0.63	0.115	0.68	0.147	0.60	0.286	0.32	0.212	0.47	0.325	0.26
G6	− 0.278	0.14	− 0.247	0.20	− 0.290	0.13	− 0.128	0.65	− 0.125	0.66	− 0.127	0.65	− 0.438	0.12	− 0.380	0.18	-0.464	0.10
G7	0.040	0.84	0.017	0.93	0.054	0.78	0.007	0.98	0.020	0.94	− 0.001	1.00	0.161	0.58	0.117	0.69	0.184	0.53
G8	0.218	0.26	0.150	0.44	0.255	0.18	0.134	0.64	0.050	0.86	0.180	0.52	0.255	0.38	0.157	0.59	0.310	0.28
G9	− 0.337	0.07	− 0.296	0.12	− 0.354	0.06	− 0.204	0.47	− 0.141	0.62	− 0.237	0.40	− 0.468	0.09	− 0.415	0.14	− 0.489	0.08
G10	0.340	0.07	0.332	0.08	0.337	0.07	0.319	0.25	0.357	0.19	0.289	0.30	0.361	0.21	0.280	0.33	0.403	0.15
G11	− 0.064	0.74	− 0.046	0.81	− 0.074	0.70	− 0.043	0.88	− 0.064	0.82	− 0.029	0.92	− 0.157	0.59	− 0.121	0.68	− 0.176	0.55
G12	− 0.316	0.10	− 0.277	0.15	− 0.332	0.08	− 0.260	0.35	− 0.308	0.27	− 0.226	0.42	− 0.406	0.15	− 0.266	0.36	− 0.483	0.08
G13	0.249	0.19	0.193	0.32	0.278	0.15	0.304	0.27	0.253	0.36	0.327	0.23	0.140	0.63	0.039	0.89	0.200	0.49
G14	− 0.364	0.05	− 0.311	0.10	− 0.388	0.04*	− 0.327	0.23	− 0.284	0.31	− 0.345	0.21	− 0.372	0.19	− 0.273	0.35	− 0.426	0.13
G15	0.256	0.18	0.310	0.10	0.217	0.26	0.249	0.37	0.331	0.23	0.194	0.49	0.331	0.25	0.363	0.20	0.304	0.29
G16	− 0.196	0.31	− 0.245	0.20	− 0.161	0.40	− 0.151	0.59	− 0.224	0.42	− 0.103	0.72	− 0.325	0.26	− 0.350	0.22	− 0.301	0.30
G17	0.105	0.59	0.101	0.60	0.105	0.59	− 0.030	0.92	− 0.062	0.83	− 0.010	0.97	0.224	0.44	0.238	0.41	0.209	0.47
G18	− 0.245	0.20	− 0.235	0.22	− 0.244	0.20	− 0.472	0.08	− 0.433	0.11	− 0.484	0.07	− 0.078	0.8	− 0.166	0.57	− 0.022	0.94
G19	− 0.307	0.11	− 0.350	0.06	− 0.272	0.15	− 0.344	0.21	− 0.390	0.15	− 0.309	0.26	− 0.177	0.54	− 0.185	0.53	− 0.167	0.57

^*^Significant *p*-value < 0.05

The significant findings of angles 14, 15, 19, 22 and 23 may be interpreted as an association between increased VAS scores and decreased lower facial third height in female models (*p* < 0.05). As ratio 5 indicates, a significantly positive association was observed between the middle facial height and VAS scores in the male models (*p* < 0.05). Ratio 17 denotes a significant association between increased VAS scores and increased alar width in females (*p* < 0.05). On the other hand, the significant results of angles 4, 9 and 10 in male models pointed to an association between an increased VAS score and a more retruded mandible, whereas the result of the canon 5 suggested a significant relationship between a narrower facial width at zygion level and an increased VAS score in females.

## Discussion

The perception of attractiveness is subjective and not constant through the years. An evident observation is a change in trends related to the ideal body shape over decades. Therefore, the ideal facial parameters should not be assumed to be constant too. Several previous studies have demonstrated that only a few suggested ‘ideal’ parameters were significantly associated with attractive [4, 7, 19–21] or norm [13, 22] faces. Some even suggested that the golden proportion does not relate at all to facial attractiveness [7, 23]. This study has found significant correlations between VAS scores and deviations of the measured parameters from ideal target values in only 14% of the overall investigated 77 parameters. This finding points out that suggested ideal values might not be applicable at all times in every population.

Some [24, 25] think that the general principle of facial aesthetics applies to both genders, whereas others [22, 26] noted distinct differences between genders. Significant results in overall facial analyses do not always appear in gender-specific subgroup analyses and vice versa. The current study has found a preference for thicker upper and lower vermillion in females but only thicker upper vermillion in males. Therefore, it is worthwhile to further investigate and develop gender-based ideal target values in the future.

This study has found a preference for a shorter lower facial third in both genders. In accordance with this finding, Mizumoto et al. (2009) [14] reported a relatively shorter lower anterior facial height among Japanese female models and actresses compared to post-orthodontic dento-skeletal class 1 patients. They further suggested a trend in aesthetic perception that favours small faces, especially small jaws. Furthermore, judges associate a shorter upper lip with attractiveness, a finding that corroborates results of other studies in the Asian population [14, 22]. Interestingly, a broader or wider nose was found to be more attractive in both genders. It may be hypothesized that this is related to the famous ‘face reading’ among the Chinese community which relates a round, fleshy and big nose to good fortune, wealth and success.

It has also been suggested that the relationship between 2-D ratios and angles and facial attractiveness is low [7]. Previous studies on facial attractiveness were mainly based on 2-D photographs [4, 5, 27] and silhouette [28] evaluations. This study has used 3-D photographs to better appreciate facial details from multiple vantage points other than just frontal and profile views. To ensure all judges were able to assess the model faces at 180°, a short video clip of a rotating 3-D photograph from the right profile view, over the frontal to the left profile views, was produce for each model. This method reduces restriction or biases compared with showing judges only 2-D frontal and profile views of photographs. It is much more realistic when compared to our daily life 3-D vision. It has been reported elsewhere [7] that the dento-skeletal pattern does not affect facial attractiveness, and one need not be class 1 to be attractive. Therefore, the models for this study comprised of dento-skeletal classes 1, 2 and 3 patterns presenting a wide range of faces with different characteristics. This explains the high variation (0 to 99.5) among the raw VAS scores recorded in this study. Additionally, only a total of thirty 3-D photographs were presented to the judges, as larger numbers might lead to their exhaustion [29], potentially affecting their assessment.

The here investigated ‘ideal’ ratios and ‘ideal’ angles are based on the recommendations of Kiekens et al. [4], 2008, who excluded parameters with low reproducible landmarks. To assess intra-assessor measurement error in this study, % TEMs of all landmarks were quantified, yielding low results, suggesting a high reproducibility of these landmarks.

This study has analysed the perception of facial attractiveness in layperson and orthognathic judges separately. It has been reported that orthognathic patients perceived their facial profile in a different way than orthodontists, surgeons and laypersons [30]. Laypersons’ perception of the facial profile was suggested to be superior to that of specialists due to the lack of any eventual professional and academic preconditioning [11]. It might be argued that therefore due to various amounts of additional specific knowledge, orthognathic patients also might differ from laypersons in their perception of attractiveness. Furthermore, as facial aesthetics is one of their main concerns, an emotional component within this specific group of patients should not be underestimated. Low satisfaction with their dento-facial appearance [30], specifically concerning certain facial features, might probably affect and influence their perception of attractiveness. Additionally, it has been reported that self-perceived attractiveness affects one’s aesthetical perception towards others [31]. Therefore, judge subgroups analyses were performed as it was assumed that orthognathic patients might present a different beauty perception than laypersons. Interestingly, this study revealed that orthognathic but not layperson judges prefer a retrusive mandible, a somehow anticipated outcome given that Chinese and Japanese are in favour of a retruded ‘soft’ mandible [14]. Besides, correlations tests have revealed several different significant results between these two groups. These results suggested that orthognathic patients apply different facial attractiveness criteria when compared to laypersons. Generally, it might be speculated that orthognathic patients put more emphasis on facial attractiveness than others. Based on self-perceived own ‘shortcoming’ facial features, they might have a specific interest related to certain facial ratios or angles. All these factors together with specific emotional and psychological components might serve to explain the different results recorded between orthognathic patient and non-orthognathic layperson judges. Therefore, patients’ wishes are of utmost importance during the orthodontic, orthognathic and/or plastic surgery treatment planning, instead of relying completely on stereotypical so-called ideal values and/or measurements. Recommended ideal values should only serve as a rough guideline in the clinical daily work. Furthermore, unique ethnic facial characteristics must be considered during all facial aesthetic treatment planning.

## Conclusion

Despite several renowned ‘ideal’ parameters of attractive faces that have been recommended in the literature, only a few of them were found to be significantly correlated with attractive faces in Hong Kong Chinese. In general, a broader inter-endocanthus and inter-exocanthus width, shorter and broader nose, shorter lower face, shorter upper lip, flatter labio-mental fold and retrusive mandible comparing to the recommended ideal faces were perceived as attractive by the judges.

## Data Availability

All data generated or analysed during this study are included in this published article.
